# Epidermal-specific deletion of TC-PTP promotes UVB-induced epidermal cell survival through the regulation of Flk-1/JNK signaling

**DOI:** 10.1038/s41419-018-0781-9

**Published:** 2018-06-28

**Authors:** Minwoo Baek, Mihwa Kim, Jae Sung Lim, Liza D. Morales, Joselin Hernandez, Srinivas Mummidi, Sarah Williams-Blangero, Ik-Soon Jang, Andrew T. Tsin, Dae Joon Kim

**Affiliations:** 1 Department of Biomedical Sciences, School of Medicine, University of Texas Rio Grande Valley, Edinburg, TX USA; 2 Department of Human Genetics, School of Medicine, University of Texas Rio Grande Valley, Edinburg, TX USA; 3 South Texas Diabetes and Obesity Institute, School of Medicine, University of Texas Rio Grande Valley, Edinburg, TX USA; 4 Division of Bioconvergence Analysis, Korea Basic Science Institute, Daejeon, 305-333 Republic of Korea; 5 Present Address: College of Pharmacy, University of Minnesota, Duluth, MN USA

## Abstract

UVB exposure can contribute to the development of skin cancer by modulating protein tyrosine kinase (PTK) signaling. It has been suggested that UVB radiation increases the ligand-dependent activation of PTKs and induces PTP inactivation. Our recent studies have shown that T-cell protein tyrosine phosphatase (TC-PTP) attenuates skin carcinogenesis induced by chemical regimens, which indicates its critical role in the prevention of skin cancer. In the current work, we report that TC-PTP increases keratinocyte susceptibility to UVB-induced apoptosis via the downregulation of Flk-1/JNK signaling. We showed that loss of TC-PTP led to resistance to UVB-induced apoptosis in vivo epidermis. We established immortalized primary keratinocytes (IPKs) from epidermal-specific TC-PTP-deficient (*K14Cre.Ptpn2*^*fl/fl*^) mice. Immortalized TC-PTP-deficient keratinocytes (TC-PTP/KO IPKs) showed increased cell survival against UVB-induced apoptosis which was concomitant with a UVB-mediated increase in Flk-1 phosphorylation, especially on tyrosine residue 1173. Inhibition of Flk-1 by either its specific inhibitors or siRNA in TC-PTP/KO IPKs reversed this effect and significantly increased cell death after UVB irradiation in comparison with untreated TC-PTP/KO IPKs. Immunoprecipitation analysis using the TC-PTP substrate-trapping mutant TCPTP-D182A indicated that TC-PTP directly interacts with Flk-1 to dephosphorylate it and their interaction was stimulated by UVB. Following UVB-mediated Flk-1 activation, the level of JNK phosphorylation was also significantly increased in TC-PTP/KO IPKs compared to control IPKs. Similar to our results with Flk-1, treatment of TC-PTP/KO IPKs with the JNK inhibitor SP600125 significantly increased apoptosis after UVB irradiation, confirming that the effect of TC-PTP on UVB-mediated apoptosis is regulated by Flk-1/JNK signaling. Western blot analysis showed that both phosphorylated Flk-1 and phosphorylated JNK were significantly increased in the epidermis of TC-PTP-deficient mice compared to control mice following UVB. Our results suggest that TC-PTP plays a protective role against UVB-induced keratinocyte cell damage by promoting apoptosis via negative regulation of Flk-1/JNK survival signaling.

## Introduction

Skin is the largest organ of the human body. Its function is to provide protection and receive stimuli from the environment; consequently, skin undergoes a great deal of environmental assault. Ultraviolet (UV) irradiation from sunlight is a principal environmental factor that can contribute to the development of all three major types of skin cancer: squamous cell carcinomas, basal cell carcinomas, and cutaneous melanomas. Of the three different ranges of UV, UVB (280–315 nm) radiation is the most significant in causing skin cancers^1–3^. Exposure to UVB radiation damages DNA, giving rise to mutations in any number of genes, including oncogenes and tumor suppressor genes, which can modulate cellular signaling pathways involved in DNA damage repair, growth, apoptosis, and differentiation. Following UVB exposure, irreparable DNA-damaged keratinocytes undergo cell death, or apoptosis, through different pathways that involve the activation of the tumor suppressor gene p53 and cell death receptor signaling. However, aberrant keratinocytes that have managed to survive or bypass apoptosis can start to proliferate during continuous UVB radiation, resulting in the development of UVB-induced skin cancer^4–6^.

T-cell protein tyrosine phosphatase (TC-PTP, encoded by *PTPN2*) is a tyrosine-specific intracellular non-receptor PTP. Members of the PTP family share a highly conserved motif that is required for dephosphorylation of their target substrate proteins, a reversible posttranslational modification that can active or deactivate proteins. PTPs have been implicated in the pathogenesis of a wide range of human diseases and have become attractive targets for clinical therapies^7^. TC-PTP is ubiquitously expressed in embryonic and adult tissues^8,9^. Alternative splicing of *PTPN2* generates two distinct forms of TC-PTP: a 45-kDa form known as TC45 (TC-PTPa) and a longer 48-kDa form known as TC48 (TC-PTPb). TC45 is targeted to the nucleus by a bipartite nuclear localization signal in its C terminus^10,11^. However, our recent studies have shown that TC45 is localized in the cytoplasm of skin keratinocytes and it is translocated to the nucleus in response to UVB irradiation via an AKT/14-3-3σ-dependent mechanism, demonstrating that tissue type is a factor in TC45 subcellular localization^12^. TC48 is a minor form of TC-PTP that is targeted to the endoplasmic reticulum by its hydrophobic C terminus^10,11^. TC-PTP modulates various cellular functions, including cell cycle regulation, proliferation, and apoptosis. TC-PTP has been well-studied due to its critical role in the regulation of diabetes and obesity through its ability to modulate insulin and leptin signaling^13^. For example, neuronal cell-specific TC-PTP-deficient mice showed reduced high-fat-diet-induced weight gain and enhanced leptin sensitivity in the hypothalamus with increased STAT3 phosphorylation after leptin administration, indicating that TC-PTP is involved in the development of leptin resistance via STAT3^14^.

Our work has revealed that TC-PTP is also essential to the skin response to UVB radiation or a two-stage chemical regimen which consists of the carcinogens 7,12-dimethylbenz[a]anthracene (DMBA) and 12-O-tetradecanoylphorbol-13-acetate (TPA). Initial studies of PTPs in skin showed that PTP expression is induced during keratinocyte proliferation and maturation, but expression levels remain unchanged within epidermal tissue^15^. It has been demonstrated that exposure to acute UV irradiation or treatment with the tumor promoter TPA increases the activation of protein tyrosine kinases, including the epidermal growth factor receptor (EGFR) and the downstream STAT3 signaling pathway^16–20^. However, we showed that STAT3 is initially dephosphorylated in keratinocytes in the early response to UVB irradiation, and treatment with sodium orthovanadate (Na_3_VO_4_), a pan PTP inhibitor, recovered the level of phosphorylated STAT3^19^. Further investigation revealed that TC-PTP is an important regulator of STAT3 and it negatively regulates STAT3-mediated survival signaling during the response to UVB radiation, which protects against proliferation of UV-damaged keratinocytes^12,21^. Our in vivo studies demonstrated that TC-PTP also regulates cell survival and apoptosis via STAT3 and AKT during DMBA/TPA-induced skin tumor formation^22^.

Vascular endothelial growth factors (VEGFs) are critical regulators for vascular development both in normal and disease conditions. The effects of VEGF are mediated by its cognate receptors (VEGFRs) and co-receptors. Binding of VEGF to its receptor induces receptor dimerization and subsequent activation through autophosphorylation of tyrosine residues located in its intracellular domains, which can trigger various downstream signaling pathways. While VEGFRs are essential for endothelial cell function, including angiogenesis, they are also expressed in various tissues such as skin, heart, and kidney^23^. In particular, all five VEGF receptors are expressed in epidermal keratinocytes^24^. Among them, Flk-1 (fetal liver kinase-1, also known as VEGFR2) was found to regulate keratinocyte proliferation and migration^25^. In addition, studies showed that expression and phosphorylation of Flk-1/VEGFR2 is increased by a moderate dose of UVB and its activation promotes keratinocyte survival upon UVB exposure^26^. Ligand-mediated activation of Flk-1/VEGFR2 leads to the activation of the mitogen-activated protein kinases extracellular signal-regulated kinase (ERK) and c-Jun N-terminal kinase (JNK) in endothelial cells^27,28^.

The JNK signaling pathway is a well-studied pathway that is an important component of mitogen-activated protein kinase (MAPK) signal transduction which includes ERK and p38 MAPK. JNK is involved in the regulation of many cellular functions, including cell proliferation and apoptosis^29,30^. In particular, it is critical in apoptosis because JNKs respond to a variety of harmful external stimuli such as UV radiation, oxidative stress, inflammation, and DNA damage and JNK signaling mediates p53 activation^31–34^. Not surprisingly, JNK signaling contributes to the pathogenesis of a number of human diseases such as diabetes, neurodegenerative disorders, and cancer, including skin cancer^35^. In our current study, we demonstrate for the first time that TC-PTP promotes apoptosis in UVB-damaged keratinocytes via inhibition of Flk-1/JNK signaling.

## Results

### Loss of epidermal TC-PTP leads to increased resistance to UVB-induced apoptosis

**Fig. 1:**
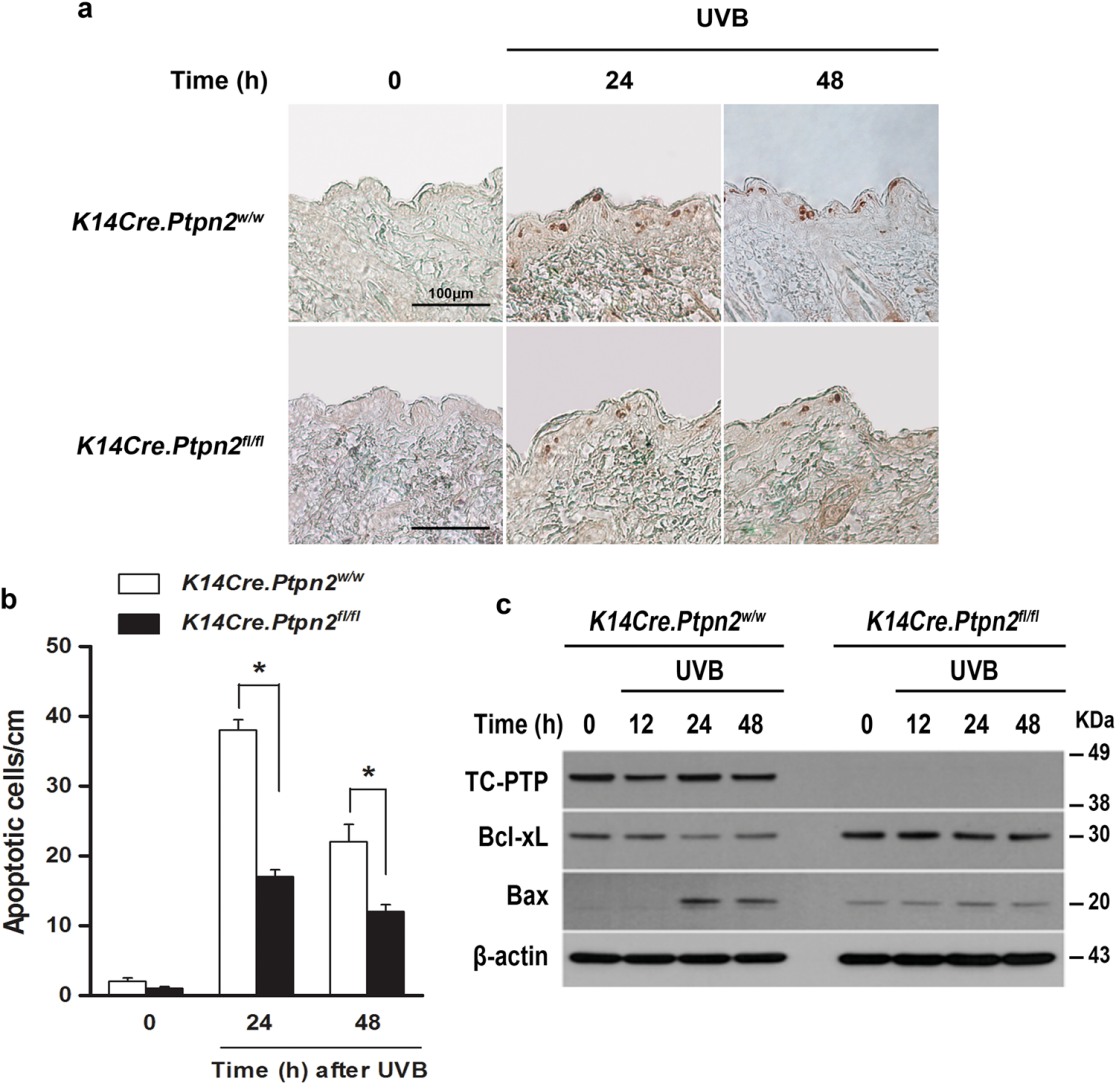
Effect of TC-PTP deletion on UVB-induced apoptosis. **a–b** Apoptotic response of the epidermis from *K14Cre.Ptpn2*^*w/w*^ and *K14Cre.Ptpn2*^*fl/fl*^ mice. Groups of mice (*n* = 3) were irradiated with UVB at 120 mJ/cm^2^ and sacrificed 24 and 48 h after irradiation. Skin sections from each mouse were collected and evaluated to measure apoptosis by immunostaining with caspase-3. **a** Representative phase-contrast image depicting active caspase-3 (dark spots) in the epidermis from both mouse genotypes before and after UVB irradiation. Scale bar: 100 μm. **b** Quantitative analysis of caspase-3-positive cells per centimeter of epidermis in both genotypes after UVB irradiation. *P < 0.05 by Mann–Whitney *U* test. **c** Western blot analysis of Bcl-xL and Bax in the epidermis from both genotypes following UVB irradiation at 120 mJ/cm^2^. Mice were sacrificed at the indicated time after UVB exposure and the total epidermal cell lysates were prepared.

We have shown that TC-PTP deficiency in mouse 3PC keratinocytes suppresses UVB-induced apoptosis by regulating STAT3 signaling and loss of TC-PTP increases resistance against DMBA-induced apoptosis in TC-PTP knockout mice^22^. In the current work, we utilized our *K14Cre.Ptpn2*^*w/w*^ (TC-PTP/WT) and *K14Cre.Ptpn2*^*fl/f*^ (TC-PTP/KO) mice to further investigate the effect of TC-PTP deficiency in UVB-induced epidermal apoptosis. As shown in Fig. 1a, b, the number of apoptotic cells, detected by staining with active caspase-3, was significantly increased in the epidermis of TC-PTP/WT mice compared to TC-PTP/KO mouse epidermis. Consistent with this observation, the level of antiapoptotic Bcl-xL expression was higher in the epidermis of TC-PTP/KO mice compared to TC-PTP/WT mice in the absence or presence of UVB irradiation, whereas the level of proapoptotic Bax expression was lower in TC-PTP/KO mice compared to TC-PTP/WT mice following UVB exposure (Fig. 1c). These results further confirm a protective function for TC-PTP in the cellular response to UVB irradiation, which ensures the elimination of UV-damaged keratinocytes.

### Generation of immortalized primary keratinocytes (IPKs) from epidermal-specific Ptpn2 knockout mice

**Fig. 2:**
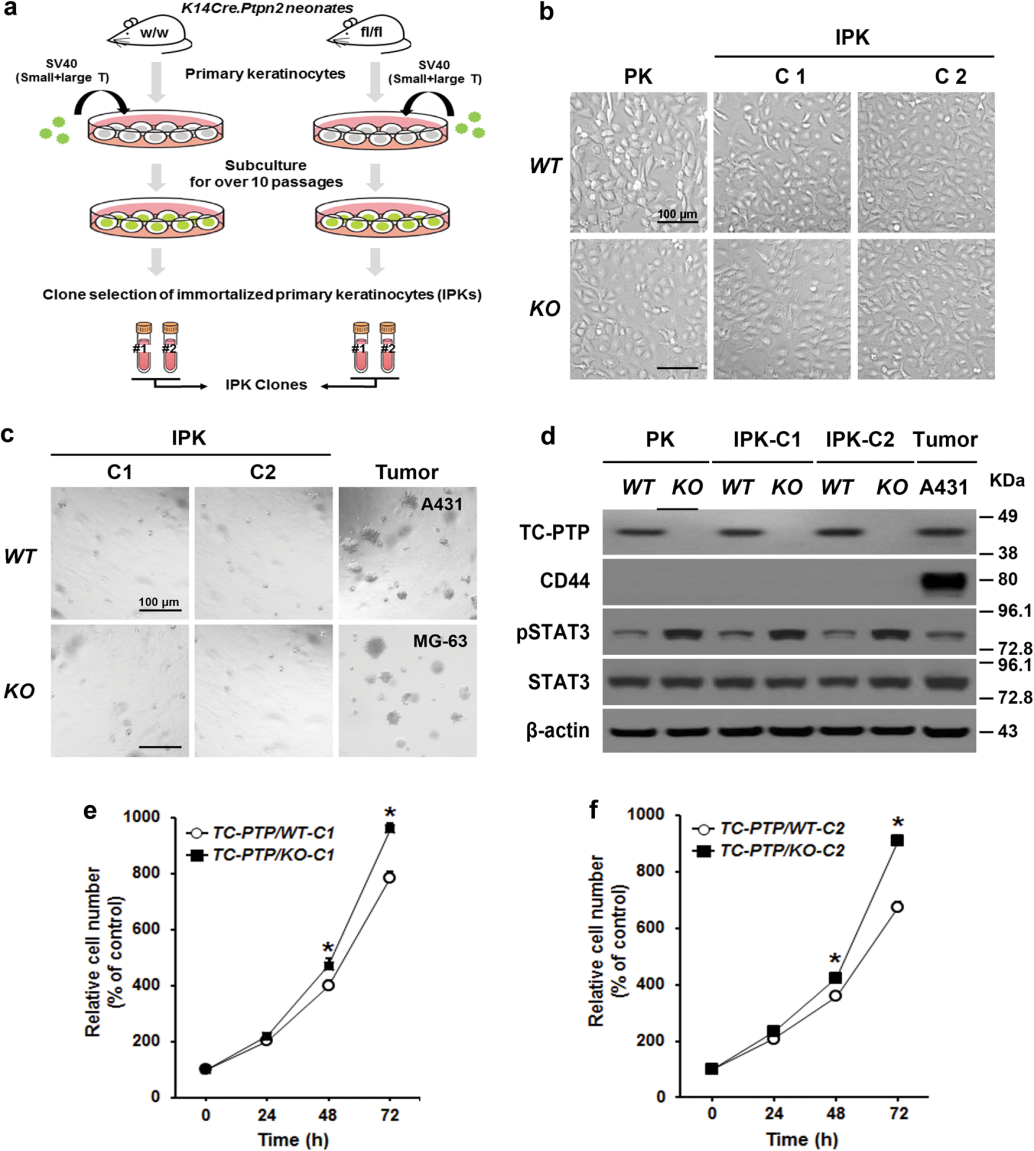
Generation and characterization of immortalized primary keratinocytes (IPKs) from *K14Cre.Ptpn2*^*w/w*^ and *K14Cre.Ptpn2*^*fl/fl*^ mice. **a** Schematic diagram of the generation of IPK lines from *K14Cre.Ptpn2*^*w/w*^ and *K14Cre.Ptpn2*^*fl/fl*^ mice using SV40 small + large T antigen. Two IPK clones (IPK-C1 and IPK-C2) were selected from each genotype, respectively. **b** Representative photomicrographs of primary keratinocytes and IPKs from both genotypes after 3 days of culture. PK: primary keratinocytes. Scale bar: 100 μm. **c** Representative photomicrographs of IPKs from both genotypes in soft agar colony formation assay after 2 weeks of culture. The human epidermoid carcinoma cell line A431 and the human osteosarcoma cell line MG-63 were used as positive controls. Scale bar: 100 μm. **d** Western blot analysis of pSTAT3, STAT3, and CD44 in primary keratinocytes and IPKs from both genotypes. The A431 cells were used as positive controls. **e–f** Proliferation of IPKs was measured using WS-1 assay, according to the manufacturer’s manual. The results are the mean ± standard deviation from three independent experiments. *P *<* 0.05 by *T* test for equality of means. **e** IPK clone C1. **f** IPK clone C2.

Mouse primary keratinocytes have been used to identify signaling mechanisms that occur in skin, but maintaining a primary culture has limitations due to a short lifespan and difficulty of subculture^36^. Therefore, we established immortalized primary keratinocytes (IPKs) from either TC-PTP/WT or TC-PTP/KO mice in order to further investigate the role of TC-PTP in UVB-induced apoptosis in vitro. Briefly, primary keratinocytes from both genotypes of mice were cultured and infected with SV40 small and large T antigen (see Materials and Methods) (Fig. 2a). Then, two clones (IPK-C1 and IPK-C2) from each genotype were selected. When the IPK clones were cultured and compared with their corresponding primary keratinocytes, no visible morphological changes were found in the IPK clones (Fig. 2b). We evaluated the anchorage-independent potential of the IPKs by using a soft agar colony formation assay. As shown in Fig. 2c, both clones were unable to form colonies, while A431 epidermoid carcinoma cells and MG-63 osteosarcoma cells, which were used as positive control, formed colonies in soft agar after 2 weeks. CD44 is one epidermal stem cell marker and keratinocytes positive for CD44 expression show enhanced colony formation^37^. Consistent with the results from the soft agar assay, IPK clones did not express CD44, while A431 cells showed its expression (Fig. 2d). We previously showed that the level of phosphorylated STAT3 was higher in the epidermis of TC-PTP/KO mice compared to that of control mice^22^. Similar with previous results, the level of phosphorylated STAT3 was higher in TC-PTP/KO primary keratinocytes and TC-PTP/KO IPK clones than TC-PTP/WT primary keratinocytes and TC-PTP/WT IPK clones, respectively. Also, the phosphorylated STAT3 expression levels were comparable between primary keratinocytes and IPK clones (Fig. 2d). Furthermore, TC-PTP/KO IPK clones significantly grew faster than the corresponding control clones (Fig. 2e, f), which is similar to the previous results using primary keratinocytes^22^. These results indicate that the IPK clones possess similar growth and morphological characteristics to the primary keratinocytes from which they were derived. Since both IPK clones C1 and C2 showed similar properties, we utilized IPK-C2 for subsequent experiments.

### TC-PTP deficiency in IPKs suppresses apoptosis following UVB irradiation

**Fig. 3:**
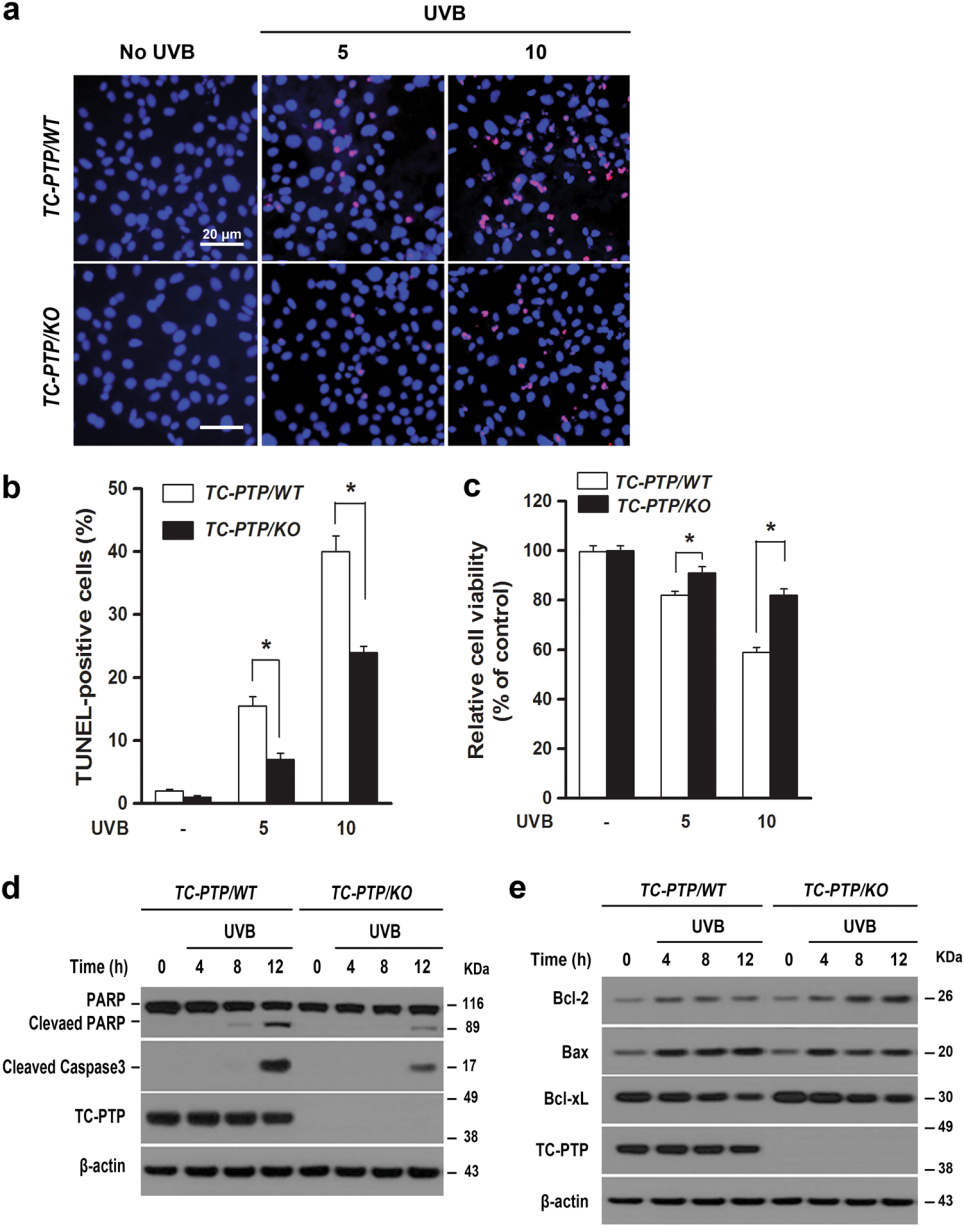
Apoptotic response of IPKs derived from the epidermis of *K14Cre.Ptpn2*^*w/w*^ and *K14Cre.Ptpn2*^*fl/fl*^ mice after UVB irradiation. **a–c** TC-PTP/WT-C2 and TC-PTP/KO-C2 were irradiated with UVB at doses of 5 or 10 mJ/cm^2^ and incubated for 18 h following UVB irradiation. **a** Representative TUNEL staining (red) from TC-PTP/WT-C2 and TC-PTP/KO-C2 after UVB exposure. Nuclei were stained with DAPI (blue). Scale bar, 20 µm. **b** Quantitative analysis of the percentage of TUNEL-positive cells from TC-PTP/WT-C2 and TC-PTP/KO-C2 IPKs. The results are the mean ± standard deviation from three independent experiments. *P < 0.05 by *T* test for equality of means. **c** Cell viability of IPKs was measured using WST-1 assay. *P *<* 0.05 by *T* test for equality of means. **d** Western blot analysis of cleaved PARP and cleaved caspase 3 in IPKs from both genotypes. Cells were collected at the indicated time after UVB irradiation (10 mJ/cm^2^) and total cell lysates were prepared. **e** Western blot analysis of the mitochondrial apoptotic marker Bcl-xL, Bcl-2, or Bax in IPKs from both genotypes. Cells were collected at the indicated time after UVB irradiation (10 mJ/cm^2^) and total cell lysates were prepared.

To investigate the impact of TC-PTP deficiency in UVB-induced apoptosis, both TC-PTP/WT and TC-PTP/KO IPKs were cultured and irradiated with UVB. Consistent with in vivo results, apoptotic cells detected by TUNEL staining were significantly increased in TC-PTP/WT IPKs compared to TC-PTP/KO IPKs which corresponded with a significant reduction in cell viability in TC-PTP/WT IPKs compared to TC-PTP/KO IPKs (Fig. 3a–c). Western blot analysis with the apoptotic markers PARP1 (poly[ADP-ribose] polymerase) and caspase-3 confirmed these results, as evidenced by a significant increase in both cleaved PARP and cleaved caspase-3 in TC-PTP/KO IPKs compared to TC-PTP/WT IPKs following UVB irradiation (Fig. 3d). In addition, the levels of Bcl-xL and Bcl-2 expression were higher in TC-PTP/KO IPKs compared to TC-PTP/WT IPKs in the absence or presence of UVB irradiation, whereas the level of Bax expression was lower in TC-PTP/KO IPKs compared to TC-PTP/WT IPKs (Fig. 3e). These results confirm that TC-PTP is a critical factor in the induction of apoptosis in keratinocytes upon UVB exposure.

### TC-PTP promotes UVB-induced apoptosis in keratinocytes by dephosphorylating Flk-1 tyrosine residue 1173

As previously mentioned, activation of Flk-1/VEGFR2 can stimulate keratinocyte survival in response to UVB irradiation^26^. TC-PTP has been shown to negatively regulate endothelial cell proliferation and migration by directly dephosphorylating Flk-1/VEGFR2^38^. These findings imply that Flk-1 may be a potential target of TC-PTP in UVB-induced apoptosis in keratinocytes. Based on this hypothesis, we first examined Flk-1 phosphorylation in both TC-PTP/WT and TC-PTP/KO IPKs.

**Fig. 4:**
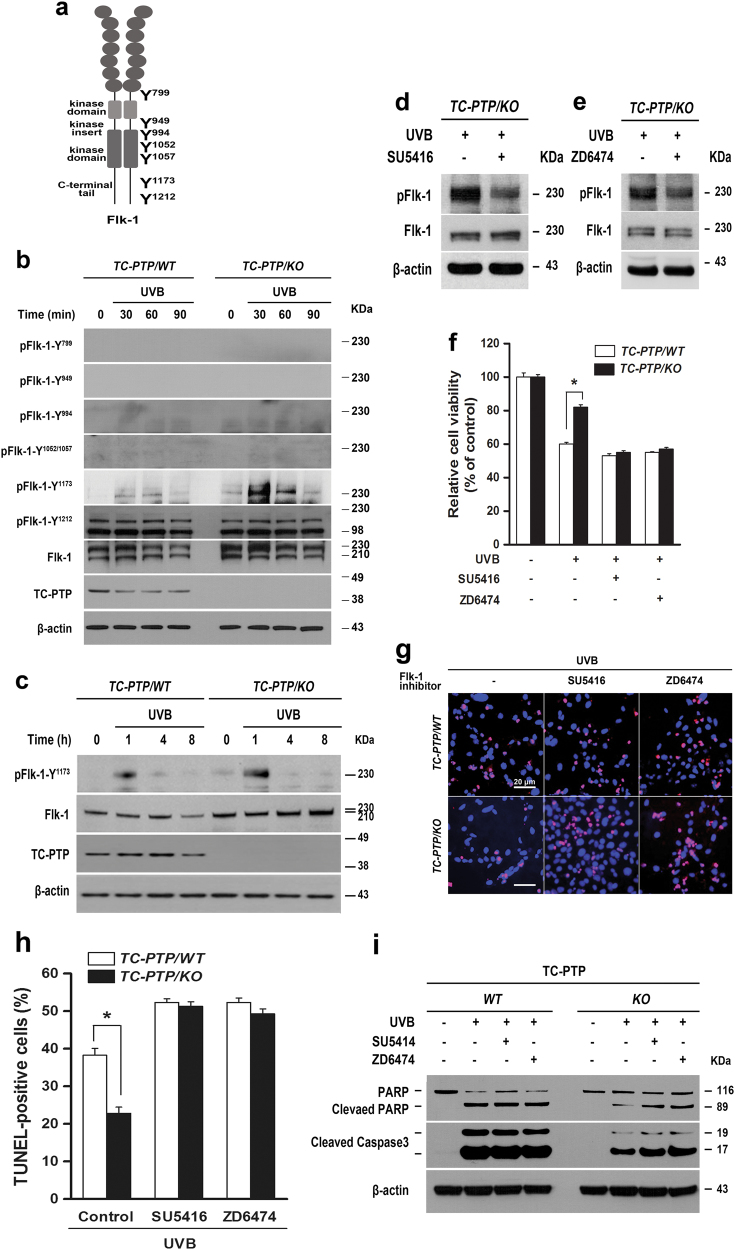
Dephosphorylation of Flk-1 by TC-PTP increases the sensitivity to UVB-induced apoptosis in keratinocytes. **a** Seven potential tyrosine phosphorylation sites of Flk-1. **b** Western blot analysis of Flk-1 tyrosine phosphorylation at seven different sites in IPKs from both genotypes. Cells were collected at the indicated time after UVB irradiation (10 mJ/cm^2^). Total cell lysates were isolated and resolved by SDS-PAGE and immunoblotted with antibodies specific for pFlk-1 Y799, Y949, Y994, Y1025/1057, Y1173, or Y1212. **c** Western blot analysis of pFlk-1 (Y1173) in IPKs from both genotypes. Cells were collected at the indicated time after UVB irradiation (10 mJ/cm^2^). Total cell lysates were isolated and analyzed. **d–e** Western blot analysis of pFlk-1 (Y1173) in TC-PTP/KO IPKs in response to UVB irradiation. TC-PTP/KO IPKs were incubated with Flk-1 inhibitors, (**d**) SU5416 (2 µM) or (**e**) ZD6474 (2 µM) for 1 h before UVB irradiation (10 mJ/cm^2^). Cells were incubated with an inhibitor again for 1 h following UVB irradiation at which time the total cell fractions were extracted. **f** Effect of Flk-1 inhibition on cell viability after UVB exposure. TC-PTP/WT IPKs and TC-PTP/KO IPKs were cultured with SU5416 (2 µM) or ZD6474 (2 µM) for 1 h before UVB irradiation (10 mJ/cm^2^). Cell viability was analyzed by WST-1 assay 18 h after UVB irradiation. The results are the mean ± standard deviation from three independent experiments. *P < 0.05 by *T* test for equality of means. **g–h** TC-PTP/WT IPKs and TC-PTP/KO IPKs were cultured with SU5416 (2 µM) or ZD6474 (2 µM) for 1 h before UVB irradiation (10 mJ/cm^2^). Apoptotic cells were analyzed by TUNEL assay 18 h after UVB irradiation. **g** Representative TUNEL staining (red) from TC-PTP/WT IPKs and TC-PTP/KO IPKs after UVB exposure. Nuclei were stained with DAPI (blue). Scale bar, 20 µm. **h** Quantitative analysis of the percentage of TUNEL-positive cells from TC-PTP/WT IPKs and TC-PTP/KO IPKs. The results are the mean ± standard deviation from three independent experiments. *P < 0.05 by *T* test for equality of means. **i** Western blot analysis of cleaved PARP and cleaved caspase 3 in TC-PTP/WT IPKs and TC-PTP/KO IPKs. IPKs from both genotypes were cultured with SU5416 (2 µM) or ZD6474 (2 µM) for 1 h before UVB irradiation (10 mJ/cm^2^). Cells were collected at 12 h after UVB irradiation.

There are seven potential tyrosine (Y) autophosphorylation sites in the Flk-1 intracellular domain^39,40^. These sites include Y799 (Y801 in human), Y949, Y994, Y1052, Y1057, Y1173, and Y1212 (Fig. 4a). Among these tyrosine phosphorylation sites, Y1173 phosphorylation was significantly increased in TC-PTP/KO IPKs compared to TC-PTP/WT IPKs 1 h after UVB irradiation, whereas phosphorylation of the other six tyrosine sites was neither expressed in keratinocytes, nor induced by UVB exposure (Fig. 4b, c). The results indicate that TC-PTP is required for dephosphorylation of Flk-1 at Y1173. To examine whether Flk-1 phosphorylation on Y1173 is involved in UVB-induced apoptosis, IPKs were treated with Flk-1-specific inhibitors, SU5416 and ZD6474, before UVB irradiation. Pretreatment with either SU5416 or ZD6474 before UVB exposure effectively reduced the level of phosphorylated Flk-1 in TC-PTP/KO IPKs (Fig. 4d, e). Next, we examined the effects of the Flk-1 inhibitors on cell viability after UVB exposure. Pretreatment with Flk-1 inhibitors before UVB exposure reduced cell viability in both TC-PTP/WT and TC-PTP/KO IPKs in comparison with untreated controls. However, the reduction in cell viability of TC-PTP/KO IPKs was higher compared to that in TC-PTP/WT IPKs (Fig. 4f). Consistent with this result, pretreatment with Flk-1 inhibitors before UVB exposure significantly increased TUNEL-positive apoptotic cells in TC-PTP/KO IPKs to a level comparable with that in TC-PTP/WT IPKs (Fig. 4g, h). Western blot analysis showed that the levels of both cleaved PARP and cleaved caspase-3 expression were increased in TC-PTP/KO IPKs following pretreatment with Flk-1 inhibitors, while their expression levels in TC-PTP/WT IPKs were unaffected (Fig. 4i).

**Fig. 5:**
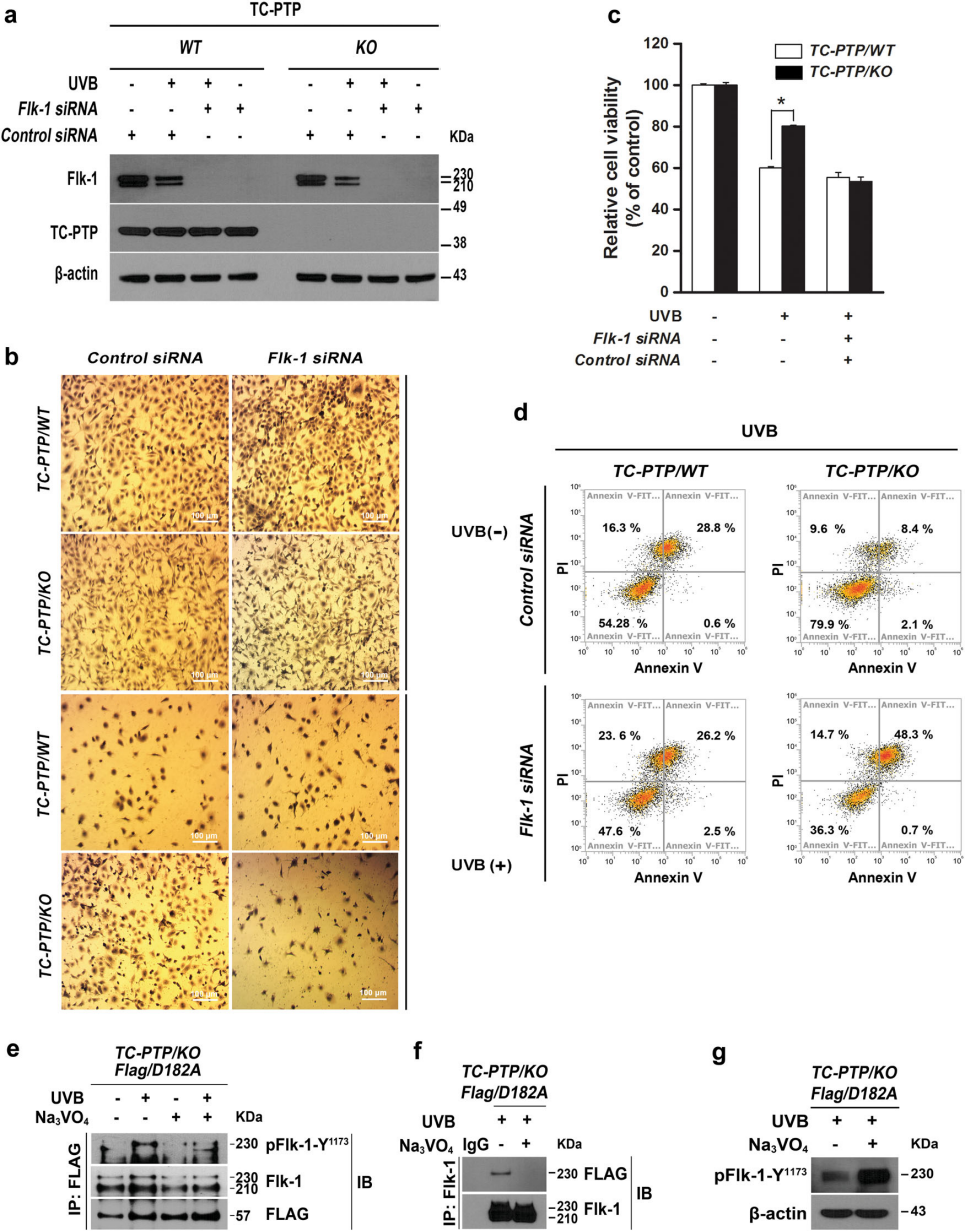
Inhibition of Flk-1 by siRNA in IPKs on the regulation of UVB-induced apoptosis. **a** TC-PTP/WT and TC-PTP/KO IPKs were cultured and transfected with siRNA specific for Flk-1. After 48 h of siRNA transfection, cells were irradiated with UVB (10 mJ/cm^2^) and then collected 1 h after UVB irradiation. **b–d** IPKs from both genotypes were cultured and transfected with control or Flk-1 siRNA. After 48 h of siRNA transfection, cells were irradiated with UVB (10 mJ/cm^2^) and then collected 18 h after UVB irradiation. **b** Representative photomicrographs of IPKs from both genotypes treated with control or Flk-1 siRNA 18 h after UVB exposure. Scale bar: 100 μm. **c** Cell viability was analyzed by WST-1 assay 18 h after UVB irradiation. The results are the mean ± standard deviation from three independent experiments. *P < 0.05 by *T* test for equality of means. **d** Quantification of apoptotic cells in control or Flk-1 siRNA-treated IPKs from both genotypes 18 h after UVB exposure. Apoptotic cells were stained with Annexin V-FITC and analyzed by using flow cytometry. **e**–**f** Interaction of TC-PTP with Flk-1 in keratinocytes following UVB irradiation. TC-PTP/KO IPKs overexpressing the substrate-trapping mutant TC-PTP/D182A with FLAG tag cultured either in the presence or absence of Na_3_VO_4_ (50 µM) for 2 h and irradiated with UVB (10 mJ/cm^2^). After 1 h of UVB irradiation, total cell lysates were harvested and subjected to immunoprecipitation with either (**e**) anti-FLAG antibody or (**f**) anti-Flk-1 antibody, resolved by SDS-PAGE, and immunoblotted with anti-FLAG, anti-Flk-1, or anti-pFlk-1 Y1173 antibodies. **g** Enhanced phosphorylation of Flk-1 (Y1173) by PTP inhibition. TC-PTP/KO IPKs overexpressing TC-PTP/D182A with FLAG tag cultured either in the presence or absence of Na_3_VO_4_ (50 µM) for 2 h before UVB irradiation (10 mJ/cm^2^). Then, cells were collected 1 h after UVB exposure. Total cell lysates were isolated and resolved by SDS-PAGE and immunoblotted with antibodies specific for pFlk-1 Y1173.

To further investigate the effect of Flk-1 activation on UVB-induced apoptosis, IPKs from TC-PTP/WT and TC-PTP/KO mice were transiently transfected with Flk-1-specific siRNA or scrambled control siRNA (Fig. 5a). Knockdown of Flk-1 in TC-PTP/KO IPKs significantly reduced cell viability and increased apoptosis compared to TC-PTP/KO IPKs transfected with control siRNA after UVB irradiation (Fig. 5b–d). Interestingly, cell viability was lower in Flk-1 siRNA-transfected TC-PTP/KO IPKs compared to similarly transfected TC-PTP/WT IPKs after UVB irradiation (Fig. 5c). In addition, more apoptotic cells were detected in Flk-1 siRNA-transfected TC-PTP/KO IPKs compared to similarly transfected TC-PTP/WT IPKs after UVB irradiation (Fig. 5d).

To confirm whether TC-PTP directly dephosphorylates Flk-1, the substrate-trapping mutant TC-PTP/D182A, which lacks phosphatase activity, was produced by substituting its essential catalytic residue aspartic acid (D) to alanine (A) and then, a TC-PTP/KO IPK cell line that overexpresses TC-PTP/D182A with a FLAG epitope tag was generated by lentiviral transduction. The mutant TC-PTP/D182A was immunoprecipitated with an anti-FLAG antibody from TC-PTP/D182A-overexpressing TC-PTP/KO IPKs and probed with an anti-Flk-1 antibody. As shown in Fig. 5e, interaction of TC-PTP with Flk-1 was increased following UVB irradiation. Pretreatment with Na_3_VO_4_ before UVB exposure blocked their interaction. Immunoprecipitation of Flk-1 in the presence or absence of Na_3_VO_4_ confirmed its interaction with TC-PTP after UVB irradiation (Fig. 5f). Consistent with these results, the level of phosphorylated Flk-1 at Y1173 was significantly increased after UVB exposure in the presence of Na_3_VO_4_ (Fig. 5g). Together, these results indicate that TC-PTP promotes an apoptotic response in keratinocytes upon UVB irradiation by directly dephosphorylating Y1173 in Flk-1.

### JNK is a downstream signal of Flk-1 that is responsible for UVB-induced keratinocyte survival

**Fig. 6:**
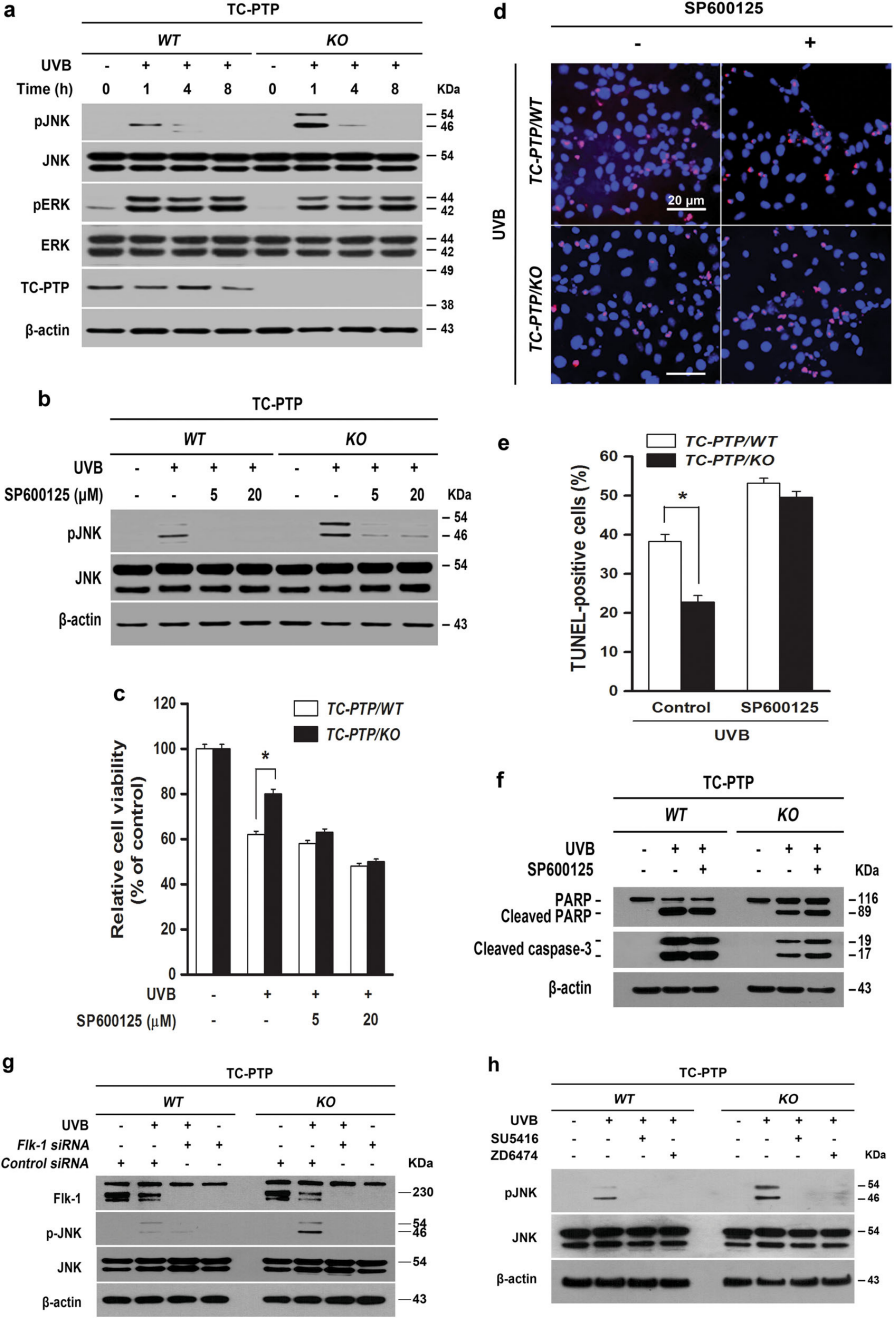
JNK is a downstream signaling target of Flk-1 in the regulation of UVB-induced apoptosis. **a** Western blot analysis of pJNK and pERK in IPKs from TC-PTP/WT and TC-PTP/KO. Cells were collected at the indicated time after UVB exposure (10 mJ/cm^2^), lysed, and subjected to SDS-PAGE gel. **b** Western blot analysis of pJNK in IPKs from both genotypes in response to UVB irradiation. TC-PTP/KO IPKs were incubated with the JNK inhibitor, SP600125 (5 or 20 µM) for 1 h before UVB irradiation (10 mJ/cm^2^). Cells were incubated with an inhibitor again for 1 h following UVB irradiation at which time the total cell fractions were extracted. **c** Effect of JNK inhibition on cell viability after UVB exposure. TC-PTP/WT IPKs and TC-PTP/KO IPKs were cultured with SP600125 (5 or 20 µM) for 1 h before UVB irradiation (10 mJ/cm^2^). Cell viability was analyzed by WST-1 assay 18 h after UVB irradiation. The results are the mean ± standard deviation from three independent experiments. *P < 0.05 by *T* test for equality of means. **d–e** TC-PTP/WT IPKs and TC-PTP/KO IPKs were cultured with SP600125 (20 µM) for 1 h before UVB irradiation (10 mJ/cm^2^). Apoptotic cells were analyzed by TUNEL assay 18 h after UVB irradiation. **d** Representative TUNEL staining (red) from TC-PTP/WT IPKs and TC-PTP/KO IPKs after UVB exposure. Nuclei were stained with DAPI (blue). Scale bar, 20 µm. **e** Quantitative analysis of the percentage of TUNEL-positive cells from TC-PTP/WT IPKs and TC-PTP/KO IPKs. The results are the mean ± standard deviation from three independent experiments. *P < 0.05 by *T* test for equality of means. **f** Western blot analysis of cleaved PARP and cleaved caspase 3 in TC-PTP/WT IPKs and TC-PTP/KO IPKs. IPKs from both genotypes were cultured with SP600125 (20 µM) for 1 h before UVB irradiation (10 mJ/cm^2^). Cells were collected at 12 h after UVB irradiation. **g–h** Western blot analysis for pJNK in IPKs from both genotypes after UVB exposure. **g** IPKs from both genotypes were cultured and transfected with control or Flk-1 siRNA. After 48 h of siRNA transfection, cells were irradiated with UVB (10 mJ/cm^2^) and then collected 1 h after UVB irradiation. **h** IPKs from both genotypes were incubated with SU5416 (2 µM) or ZD6474 (2 µM) for 1 h before UVB irradiation (10 mJ/cm^2^). Cells were incubated with an inhibitor again for 1 h following UVB irradiation at which time the total cell fractions were extracted.

In keratinocytes, UVB irradiation can initially stimulate ERK and JNK activation to protect cells against UVB-induced apoptosis^41,42^, which implies that ERK or JNK are potential downstream targets of TC-PTP/Flk-1. Therefore, we examined the levels of phosphorylated ERK and phosphorylated JNK in TC-PTP/WT and TC-PTP/KO IPKs in response to UVB irradiation. Both phosphorylated ERK and phosphorylated JNK expression levels were increased after UVB irradiation (Fig. 6a). While the level of phosphorylated ERK was similarly increased in both types of cells after UVB irradiation, the level of phosphorylated JNK was significantly increased in TC-PTP/KO IPKs compared to TC-PTP/WT IPKs following UVB irradiation (Fig. 6a). To examine whether JNK is involved in UVB-induced apoptosis, IPKs were treated with the JNK-specific inhibitor SP600125 before UVB irradiation. Pretreatment with SP600125 before UVB exposure effectively reduced the level of phosphorylated JNK in both genotypes of IPKs (Fig. 6b). Pretreatment of the JNK inhibitor before UVB exposure reduced cell viability in both TC-PTP/WT and TC-PTP/KO IPKs in comparison to untreated controls. However, the reduction in cell viability of TC-PTP/KO IPKs was higher compared to that in TC-PTP/WT IPKs (Fig. 6c). Consistent with this result, pretreatment of the JNK inhibitor before UVB exposure significantly increased TUNEL-positive apoptotic cells in TC-PTP/KO IPKs to a level comparable with that in TC-PTP/WT IPKs (Fig. 6d, e), which is similar to the results we obtained using Flk-1 inhibitors (Fig. 4g, h). Western blot analysis showed that the levels of both cleaved PARP and cleaved caspase-3 expression were increased in TC-PTP/KO IPKs with pretreatment of the JNK inhibitor, while their expression levels were not changed in TC-PTP/WT IPKs (Fig. 6f). In addition, inhibition of Flk-1 expression or phosphorylation by either siRNA or inhibitors, respectively, reduced the level of phosphorylated JNK in IPKs after UVB irradiation (Fig. 6g, h), demonstrating that JNK is a downstream signaling target of Flk-1.

**Fig. 7:**
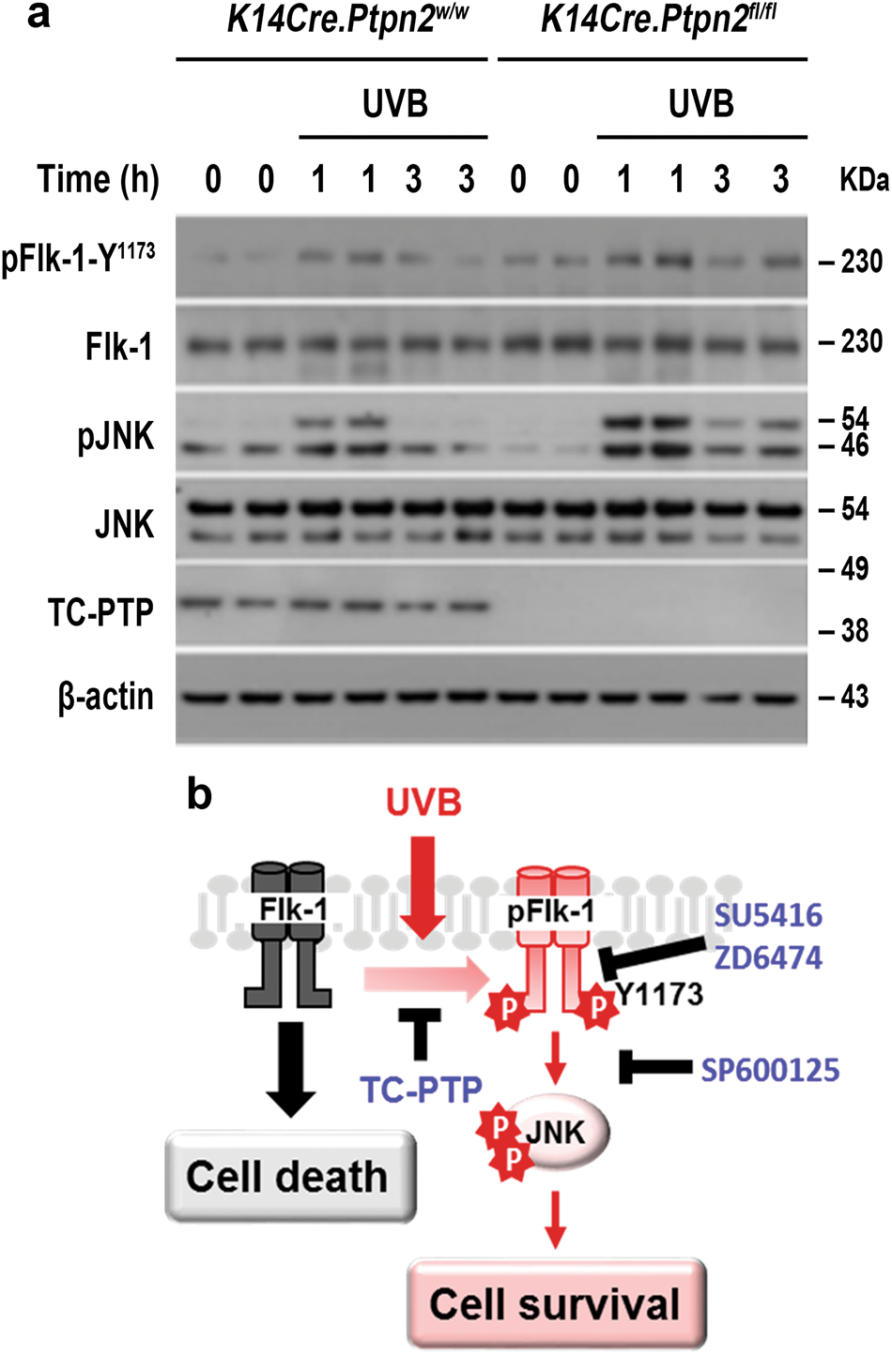
Activation of Flk-1/JNK signaling axis in the epidermis of TC-PTP- deficient mice following UVB exposure. **a** Western blot analysis of pFlk-1 (Y1173) and pJNK in the epidermis from *K14Cre.Ptpn2*^*w/w*^ and *K14Cre.Ptpn2*^*fl/fl*^ mice following UVB irradiation at 120 mJ/cm^2^. Mice were sacrificed at the indicated time after UVB exposure and total epidermal cell lysates were prepared. **b** Schematic diagram of the regulation of Flk-1/JNK signaling by TC-PTP following UVB irradiation. UVB triggers the phosphorylation of Flk-1 (Y1173) which is a direct target of TC-PTP. In the absence of TC-PTP, Flk-1 phosphorylation on Y1173 can activate JNK signaling, resulting in the promotion of UVB-induced cell survival. TC-PTP contributes to increased epidermal apoptosis in response to UVB exposure by dephosphorylating Flk-1, which may serve as part of an initial protective mechanism against UVB radiation damage.

To examine TC-PTP-mediated regulation of Flk-1/JNK signaling during the response to UVB irradiation in vivo, both TC-PTP/WT and TC-PTP/KO mice were irradiated with UVB, and epidermal cell lysates were prepared for western blot analysis. As shown in Fig. 7a, the levels of phosphorylated Flk-1 and phosphorylated JNK in the epidermis of TC-PTP KO mice were significantly increased in comparison to control mice, especially 1 h after UVB exposure. Collectively, our findings indicate that TC-PTP negatively regulates Flk-1/JNK signaling via direct dephosphorylation of Flk-1 on its Y1173 residue, which contributes to increased epidermal apoptosis in response to UVB exposure (Fig. 7b).

## Discussion

PTPs are involved in the regulation of various physiological processes, including growth, differentiation, metabolism, and motility, and their activation/inhibition have been implicated in various types of cancers including skin cancer^43,44^. Our previous study suggested that three PTPs––TC-PTP, SHP1, and SHP2––play a role in UVB-induced epidermal apoptosis and proliferation through the dephosphorylation of STAT3, a major oncogenic transcription factor in skin^12^. Further study demonstrated that TC-PTP is a more significant negative regulator of STAT3 in keratinocytes^21^. TC-PTP is mainly localized to the cytoplasm and then is translocated to the nucleus following UVB exposure, which leads to the dephosphorylation of STAT3 in the nucleus and the promotion of apoptosis^45^. In the current study, we describe an additional novel mechanism for TC-PTP-mediated regulation of UVB-induced epidermal apoptosis. UVB irradiation initially induces Flk-1 phosphorylation, particularly at Y1173 (Y1175 in human), but then TC-PTP directly dephosphorylates Flk-1, which leads to the inhibition of JNK activation and results in the promotion of UVB-induced apoptosis. Inhibition of either Flk-1 or JNK activation recovered the UVB-mediated apoptotic response that was reduced by loss of TC-PTP in TC-PTP/KO IPKs (Fig. 7b). Studies showed that Flk-1 can induce STAT3 phosphorylation in a ligand-independent manner in brain tumors^46^. This finding implies that Flk-1 may also contribute to enhanced keratinocyte survival following UVB exposure by triggering the activation of STAT3 survival signaling. Our work suggests that TC-PTP is a major regulator of UVB-induced epidermal apoptosis through its ability to dephosphorylate both Flk-1 in the cytoplasm and STAT3 in the nucleus.

Flk-1/VEGFR2 is a well-known, major VEGF signaling receptor that plays important roles in endothelial cell proliferation, migration, survival, and angiogenesis^39^. Studies showed that it is expressed in keratinocytes^24^. Flk-1 is overexpressed in psoriatic epidermal keratinocytes and its expression is regulated by calcium, independent of VEGF^47^. In particular, studies showed that Flk-1 expression and phosphorylation on Y1173, which is independent of VEGF, was increased by UVB irradiation in human keratinocytes. Inhibition of Flk-1 expression promoted UVB-induced cell apoptosis^26^. Furthermore, Mattila et al. showed that TC-PTP can attenuate endothelial cell proliferation, migration, and sprouting angiogenesis by directly dephosphorylating Flk-1^38^. Consistent with these studies, tyrosine phosphorylation of Flk-1 on Y1173 was increased in mouse keratinocytes after UVB irradiation, and TC-PTP deficiency further increased the level of phosphorylated Flk-1 (Fig. 4a, b). Additionally, we demonstrated that UVB-induced activation of Flk-1 is negatively regulated by TC-PTP through direct dephosphorylation (Fig. 5e), resulting in enhanced apoptosis (Figs. 4h and 5d). Use of a TC-PTP substrate-trapping mutant for immunoprecipitation analysis showed that TC-PTP dephosphorylates four different tyrosine residues of Flk-1––Y1052, Y1057, Y1212, and Y994––in human umbilical vein endothelial cells; however, it did not dephosphorylate the Y1173 residue of Flk-1^38^. Our results also showed that TC-PTP dephosphorylates Flk-1 in a phosphorylation site-specific manner in keratinocytes following UVB exposure. However, in contrast to endothelial cells, TC-PTP dephosphorylates only one residue, Y1173, out of seven major putative tyrosine phosphorylation sites in Flk-1 (Fig. 4b).

It is known that JNK activation is induced by extracellular stresses, such as TNFα and UV irradiation, to promote apoptosis^33,34^. However, the exact role of JNK in apoptosis is still controversial. Recent studies showed that UVB-mediated JNK activation protects keratinocytes from apoptosis by stimulating autophagy^42^, indicating that further studies are needed to elucidate the exact function and mechanism of JNK activation in terms of apoptosis. Similar with recent studies, our results showed an antiapoptotic function of JNK activation in response to UVB exposure. In the absence of TC-PTP, Flk-1-mediated activation of JNK significantly reduced UVB-mediated apoptosis. The inhibition of its activation in TC-PTP/KO cells by specific inhibitor SP600125 recovered the extent of apoptosis after UVB, which is comparable with that observed in TC-PTP/WT cells (Fig. 6d, e). Inhibition or inactivation of Flk-1 suppressed UVB-mediated JNK activation (Fig. 6g, h). UVB irradiation activates not only JNK, but also ERK in keratinocytes^41,42^. Similarly, we found that UVB irradiation induced ERK phosphorylation, but its activation was not affected by TC-PTP or TC-PTP-mediated activation of Flk-1 (Fig. 6a).

In conclusion, use of our epidermal-specific TC-PTP knockout mouse model and the generation of immortalized keratinocyte cell lines from this model allowed us to uncover a novel mechanism by which TC-PTP promotes epidermal apoptosis in response to UVB exposure. TC-PTP dephosphorylates activated Flk-1 at Y1173 after UVB irradiation, which is followed by the suppression of JNK phosphorylation. TC-PTP-mediated downregulation of Flk-1/JNK signaling leads to enhanced apoptosis in UVB-irradiated keratinocytes. Our findings indicate that TC-PTP contributes to the removal of UVB-damaged keratinocytes by promoting apoptosis via the negative regulation of Flk-1/JNK signaling, and modulation of TC-PTP regulatory signaling and/or use of TC-PTP activators is a potential therapeutic strategy for the prevention of skin carcinogenesis.

## Materials and methods

### TC-PTP knockout (*K14Cre.Ptpn2*^*fl/fl*^) mice and UVB irradiation

Development of epidermal-specific TC-PTP knockout (*K14Cre.Ptpn2*^*fl/fl*^) mice has been described previously^22^. Female transgenic and non-transgenic littermates at 7~8 weeks of age were used for the described experiments. The dorsal skin of each mouse was shaved 48 h before UVB irradiation; only those mice in the resting phase of the hair cycle were used. For UVB irradiation, Westinghouse FS20 sun lamp bulbs with a peak emission at 313 nm were used. The fluence rate was measured with an IL1400A Radiometer/Photometer coupled to a SEL240/UVB-1/TD detector (International Light, Inc., Newburyport, MA, USA). Each mouse was held in individual compartments of a plastic cage on a rotating base to prevent any influence of the differences across the UVB-light bulbs. A UVB-transparent lid covering the radiation chamber was used to filter out the small amount of UVC radiation emitted from these lamps. All experiments with mice were carried out with strict adherence to both institutional and National Institutes of Health (NIH) guidelines for minimizing distress in experimental animals, and all experiments including mice were approved by the University of Texas Rio Grande valley Institutional Animal Care and Use Committee (IACUC).

### Establishment of immortalized primary keratinocyte cell lines

Primary keratinocytes obtained from 2-day-old *K14Cre.Ptpn2*^*w/w*^ (TC-PTP/WT) or *K14Cre.Ptpn2*^*fl/fl*^ (TC-PTP/KO) neonates were cultured in keratinocyte growth medium (PromoCell, #C-20211, C-39011) containing 1% fetal bovine serum (FBS) and 1% penicillin/streptomycin at 37 °C and 5% CO_2_ according to the previously described method^48^. Mouse immortalized keratinocyte cell lines were established by using pLenti CMV/TO SV40 small + large T (Addgene, #22298). Viral packaging using 293T cells and titration of viruses were performed by using ViraPower^TM^ Lentiviral Packaging Mix (Invitrogen, K497500). The packed virus was concentrated by Lenti-X^TM^ Concentrator (Clontech, 631231). For establishing stable immortalized keratinocyte cell lines, primary keratinocyte cells were infected with lentivirus containing the pLenti SV40 small + large T-antigen-expressing vector. After transduction, cells were cultured with keratinocyte growth medium for over ten passages to select for the clones capable of growing indefinitely.

### Cell culture and UVB irradiation

Primary keratinocytes and immortalized primary keratinocytes (IPKs) obtained from 2-day-old *K14Cre.Ptpn2*^*fl/fl*^ or *K14Cre.Ptpn2*^*w/w*^ neonates were cultured in keratinocyte growth medium containing 1% FBS and 1% penicillin/streptomycin at 37 °C and 5% CO_2_ until 80–85% confluent, at which time cells were irradiated with UVB (5 or 10 mJ/cm^2^). Prior to UVB irradiation, keratinocytes were washed with DPBS. A small volume of DPBS was added to coat keratinocytes with a thin layer of DPBS, and then keratinocytes were irradiated with UVB. Immediately after irradiation, DPBS was removed, and prewarmed medium was added to cells for additional culture before harvest. Human A431 (ATCC^®^ CRL-1555™, epidermoid carcinoma) or human MG-63 (ATCC^®^ CRL-1427™, osteosarcoma) cells were cultured in DMEM containing 10% FBS.

### Soft agar colony formation assay

Soft agar colony formation assay was performed in six-well plates to evaluate anchorage-independent proliferation potential of IPKs generated from *K14Cre.Ptpn2*^*fl/fl*^ or *K14Cre.Ptpn2*^*w/w*^ mice. The bottom layer of each well was prepared with 2 ml of keratinocyte growth medium containing 0.6% low-melting-point agarose (Lonza, #50080). After plating, the bottom layer was solidified at 4 °C. The top layer of each well was prepared with 1 ml of keratinocyte growth medium containing 0.6% low-melting-point agarose and 5 × 10^4^ IPKs and then plated on the top of the bottom layer. After solidification of the top layer at 4 °C, 1 ml of keratinocyte growth medium was added to the top layer. Cells were allowed to grow at 37 °C and 5% CO_2_ for 14 days. Cells were fixed in 4% paraformaldehyde and stained with 0.005% crystal violet. The experiments were repeated in triplicate.

### Small interfering RNAs and inhibitors

Keratinocytes were grown overnight to ~40–60% confluence and transfected with control siRNA (Santa Cruz Biotechnology, sc-45924) or Flk-1-specific siRNA (Santa Cruz Biotechnology, sc-35390). Transfection was performed with Lipofectamine^®^ RNAiMAX (Invitrogen) according to the manufacturer’s instructions.

For inhibition of Flk-1 or JNK, IPK cells were preincubated with Flk-1 inhibitors (SU5416 or ZD6474) or the JNK inhibitor (SP600125) for 1 h. After UVB exposure (10 mJ/cm^2^), cells were incubated with an inhibitor again until they were collected. All three inhibitors were obtained from Selleck Chemical LLC.

### Analysis of UVB-induced apoptosis in keratinocytes and epidermis

Terminal DNA transferase-mediated dUTP nick-end labeling (TUNEL) assay was performed to investigate UVB-induced apoptosis in keratinocytes. IPKs were cultured and irradiated with UVB. After UVB irradiation, apoptotic cells were detected using In Situ Cell Death Detection Kit, Fluorescein (Roche Applied Sciences) following the manufacturer’ instructions. Apoptotic cells were using Leica DM 6000 fluorescent microscope, and data were presented as the average percentage of apoptotic cells/frame/slide examined.

To analyze UVB-induced epidermal apoptosis, groups of *K14Cre.Ptpn2*^*w/w*^ and *K14Cre.Ptpn2*^*fl/fl*^ mice (*n* = 3/group) were irradiated with UVB (120 mJ/cm^2^) and sacrificed 24 h and 48 h after irradiation. Skin sections were stained with an antibody to the active form of caspase 3 (R&D Systems) and then treated with a biotinylated anti-rabbit IgG-conjugated ABD reagent (BD Biosciences). Apoptotic keratinocytes were counted manually under phase contrast from at least three random, non-overlapping fields of view of tissue sections from three individual mice.

### Cell viability assay

Cell viability was assessed using a 2-(4-iodophenyl)-3-(4-nitrophenyl)-5-(2,4-disulfophenyl)-2H-tetrazolium, monosodium salt (WST-1) (Dojindo Laboratories, Japan) colorimetric assay. Briefly, IPKs were plated at a density of 2 × 10^4^ cells/well in a 48-well plate. Then, cells were irradiated with UVB as described above and reincubated for 24 h. Then, the formazan dye was added to each well and absorbance was measured at 450 nm.

### Establishment of the substrate-trapping mutant TC-PTP/D182A overexpressing stable cell lines

Stable IPK cell lines overexpressing TC-PTP substrate-trapping mutant (referred to as TC-PTP/D182A) were derived using IPKs derived from *K14Cre.Ptpn2*^*w/w*^ mice, as described above and established by lentiviral transduction with a modified lentiviral system based on Gateway^®^ cloning technology (Invitrogen)^49^. Wild-type mouse TC-PTP (45-kDa) cDNA from pEF-1α/pENTR B TC-PTP^21^ was amplified by PCR and cloned into the lentivirus entry vector pENTR4-FLAG (Addgene #17423). pENTR4-FLAG TC-PTP was then mutated by QuikChange^®^ site-directed mutagenesis kit (Stratagene) to generate TC-PTP/D182A using the corresponding site-specific mutant primers: TC-PTP_D182A_Fwd (TATACCACCTGGCCAGCTTTTGGGGTTCCAGAG) and TC-PTP_D182A_Rev (CTCTGGAACCCCAAAAGCTGGCCAGGTGGTATA). Gateway^®^ LR Clonase^®^ enzyme mix (Invitrogen) was used to catalyze an LR recombination reaction between the entry vectors expressing the TC-PTP/D182A mutant and the destination vector to generate the final lentiviral expression clones for the TC-PTP/D182A mutant. Then, TC-PTP/KO IPKs were infected with lentiviral particles from the lentiviral expression clones and selected with puromycin.

### Immunoprecipitation

Total cell lysates were prepared with Nonidet P-40-containing immunoprecipitation buffer (40 mM Tris, pH 7.4, 120 mM NaCl, 10 mM EDTA, and 0.1% (v/v) Nonidet P-40) containing protease inhibitor mixture, and phosphatase inhibitor cocktail I and II. Prior to immunoprecipitation, antibodies are incubated with magnetic Dynabeads™ Protein G (Invitrogen), and 2 mg of protein lysates were added into anti-FLAG (anti-FLAG^®^ M2) or anti-Flk-1-attached magnetic beads and incubated overnight at 4 °C. After immunoprecipitation, the beads were washed three times with washing buffer, and then the immune complexes were eluted from the beads and subjected to SDS-PAGE and immunoblot analysis.

### Preparation of protein lysates and western blot analysis

Total cell lysates from both keratinocytes and the epidermis were prepared with RIPA buffer (Thermo Fisher Scientific) containing 1% Triton X-100, protease inhibitor cocktail (Sigma-Aldrich), and phosphatase inhibitor cocktail I and II (Sigma-Aldrich). Equal amounts of total protein were resolved using SDS-PAGE and transferred to the PVDF membrane (GE Healthcare). The membrane was incubated overnight at 4 °C with a primary antibody, followed by incubation with a secondary antibody conjugated to horseradish peroxidase. Enhanced chemiluminescence detection reagents (Pierce) were used to detect immunoreactive protein. The following antibodies were used: anti-phospho-Flk-1 (Tyr949) (#2471); anti-phospho-Flk-1 (Tyr994) (#2474); anti-phospho-Flk-1 (Tyr1173) (#2478); anti-Flk-1 (# 9698); anti-phospho-STAT3 (#9145); anti-STAT3 (#9139); anti-phospho-ERK (#4370); anti-ERK (#4695); anti-phospho-JNK (#4668); anti-JNK (#9258); anti-Bcl-xL (#2762); anti-Bax (#2772); anti-TC-PTP (#58935); anti-cleaved caspase 3 (#9664); and anti-PARP (#9542) from Cell Signaling Technology; anti-phospho-Flk-1 (Tyr1052/1057) (#44-1047G); anti-phospho-Flk-1 (Tyr1212) (#44-1052) from Invitrogen; anti-phospho-Flk-1 (Tyr779) (#VP2921) from ECM Biosciences; anti-β-actin (sc-47778) from Santa Cruz Biotechnology; anti-FLAG^®^ M2 (#F3165) from Sigma-Aldrich; and anti-CD44 (#NBP1-47386) from Novus Biologicals.
